# Valorization of Cattle Slaughtering Industry By-Products: Modification of the Functional Properties and Structural Characteristics of Cowhide Gelatin Induced by High Hydrostatic Pressure

**DOI:** 10.3390/gels8040243

**Published:** 2022-04-14

**Authors:** Yanlei Gao, Liyuan Wang, Yi Qiu, Xiaona Fan, Li Zhang, Qunli Yu

**Affiliations:** College of Food Science and Engineering, Gansu Agriculture University, Lanzhou 730070, China; gyl19970925@163.com (Y.G.); wly04122022@163.com (L.W.); qiuyi00217@163.com (Y.Q.); qq1131692978@163.com (X.F.); douqwfan@163.com (Q.Y.)

**Keywords:** high hydrostatic pressure, cowhide, gelatin, functional characteristics, structure

## Abstract

This study investigates the effects of different pressures (200, 250, 300, 350, and 400 MPa) and durations (5, 10, 15, 20, and 25 min) on the functional properties, secondary structure, and intermolecular forces of cowhide gelatin. Our results show that high hydrostatic pressure significantly affected the two, three, and four-level structures of gelatin and caused the contents of the α-helix and β-turn to decrease by 68.86% and 78.58%, respectively (*p* < 0.05). In particular, the gelatin at 300 MPa for 15 min had the highest gel strength, emulsification, solubility, and foaming of all the treatment conditions under study. The analysis of the surface hydrophobicity, sulfhydryl content, zeta potential, and Raman spectroscopy shows that at a pressure of 300 MPa (15 min), the hydrogen bonds and hydrophobic interactions between collagen molecules are strongly destroyed, leading to changes in the tertiary and quaternary conformation of the protein and unfolding, with the electrostatic repulsion between protein particles making the decentralized state stable. In conclusion, moderate pressure and time can significantly improve the functional and structural properties of collagen, which provides theoretical support and guidance for realizing the high-value utilization of cowhide.

## 1. Introduction

Meat processing produces a lot of byproducts such as blood, bones, meat trimmings, skin, fat, horns, hooves, feet, skulls, and internal organs [1]. As one of the byproducts of cattle slaughter, cowhide accounts for about 5.1–8.5% of the live weight of cattle and contains more than 85% collagen and eight essential amino acids [2]. However, due to the limited scale of slaughter and the old-fashioned processing technology in China, most of the cowhide is discarded, which not only vastly wastes byproduct resources but also badly contaminates the environment [3].

At present, animal skin is mainly used in the form of gelatin for food, medicine, cosmetics, and photography [4]. As the main raw materials of commercial gelatin, pigskin and cowhide account for 42% and 29% of the global market for gelatin, respectively [5]. Cowhide gelatin is usually a thermoreversible protein gel prepared by the thermal denaturation and partial hydrolysis of cowhide collagen. Its melting point is close to the temperature of the human body and thus melts quickly after it enters the oral cavity [6]. So far, cowhide gelatin interacts with food macromolecules such as meat protein and whey protein and has been developed into a number of new products such as meat patties, ice cream, and yogurt [3]. The addition of cowhide gelatin can greatly enhance the textural properties and syneresis of yogurt [7], improve the water retention and cooking loss of chicken meat [8], and help impart the expected biological activity of skimmed yogurt [9]. However, Ma, Zhao, and Zhao [9] found that the addition of cowhide gelatin delayed the fermentation of skimmed yogurt, inhibited acid production during the fermentation process, and reduced the interaction between casein in yogurt, resulting in less protein gel being synthesized in yogurt, thereby reducing the texture and stability of yogurt. They speculated that this may be closely related to the covalent bonding within the cowhide gelatin, intermolecular forces, and structural properties. Hence, it is necessary to modify cowhide so that it can impart better quality characteristics to the product.

The traditional methods for extracting gelatin include acidification, alkalization, and enzyme treatment, etc., but the production cycle is long, the cost is high, the production efficiency is low, and the environment is polluted as a result [10]. At present, the application of green technologies, such as ultrasound, microwaves, high pressure, and subcritical water extraction, in the gelatin industry has become a hot topic. These technologies can not only shorten the operating time of gelatin, increase the productivity of gelatin, and improve the quality of gelatin, but they can also solve the problems of environmental pollution and waste management during the preparation of gelatin [11]. Ultrasound and subcritical water extraction alone cannot completely hydrolyze collagen. They must be combined with a catalyst to extract gelatin [11]. Microwaves cause the heat of the solvent to be transferred to the gelatin surface, which reduces the yield of gelatin [12]. Ultra-high-pressure processing is one of the most successful non-thermal processing technologies used in the food industry [13], and it mainly includes high hydrostatic pressure technology, high-pressure dynamic microfluidization, and high-pressure homogenization [14,15]. These technologies can prominently improve the protein–water and protein–protein interactions in foods, resulting in the physicochemical modification and improvement of functional properties of foods [16,17,18]. High hydrostatic pressure technology has been used in food processing and storage since 1899 and has excellent functional properties such as killing microorganisms, retaining food nutrients, and modifying structures [19]. It is reported that high hydrostatic pressure can significantly change the conformation of glycosylated soy protein isolate and improve its solubility [20] and increase the emulsifying performance of myosin [14], sweet potato protein hydrolysates [21], and common soy protein isolate [22]. Sezer, Okur, Oztop, and Alpas [23] found that high hydrostatic pressure can significantly improve the gel strength and rheological properties of fish gelatin. However, the modification of functional and structural properties of cowhide gelatin by high hydrostatic pressure technology has not yet been reported.

Based on this technology, our study investigated the influence of different durations and different pressures on the functional properties, secondary structure, and intermolecular forces of cowhide gelatin, and then speculated on the mechanism of the effect of high hydrostatic pressure on the molecular structure of collagen, which also provided a theoretical basis for the value-added technology and high-value utilization of by-products in the cattle slaughtering and processing industry.

## 2. Results and Discussion

### 2.1. Functional Characteristics

#### 2.1.1. Gel Strength

**Figure 1:**
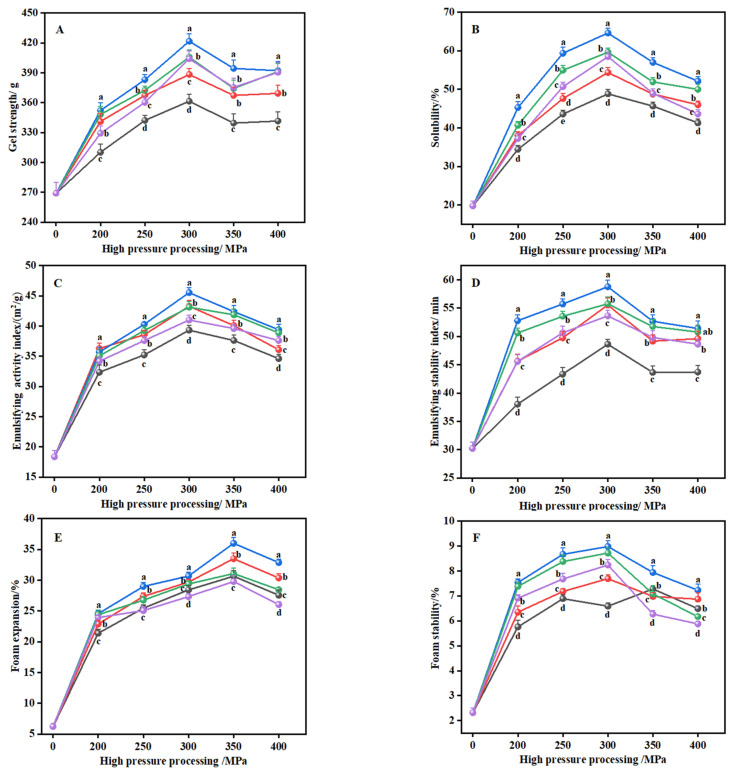
The effect of high hydrostatic pressure treatment on the gel strength (**A**), solubility (**B**), emulsification (**C**,**D**), and foaming (**E**,**F**) of cowhide collagen. The lowercase letters (a–e) indicate that different high-pressure durations have significant differences among the same pressure. High pressure 5 min (●); high pressure 10 min (●); high pressure 15 min (●); high pressure 20 min (●); high pressure 25 min (●).

As shown in Figure 1A, the gel strengths of the pressure group (310.21–421.67 g) were prominently higher than those of the control gelatin (269.03 g) (*p* < 0.05). With increasing pressure and durations, the gel strength of gelatin first rises then falls. The gel strength of the gelatin treated at 300 MPa was the highest, and the gel strength of the gelatin treated for 5 min under this pressure was the best. This is due to the fact that when treated with a certain pressure, the proteins unfold and arrange in an orderly manner to promote the formation of organized three-dimensional networks formed by covalent bonds and non-covalent cross-links, forming a gel matrix with a higher gel strength [16]. Moreover, with the increase in duration and pressure, the protein unfolds further, which enhances the interactions between unfolded proteins with more cross-linking sites and significantly increases the density of the gel matrix during the formation of the protein’s three-dimensional network structure. However, when the pressure and duration are increased above a certain level, these effects become less obvious, which may be due to the excessive expansion, repeated folding, or aggregation of proteins reducing the cross-linking degree of the three-dimensional network structure of the gel, resulting in a decrease in the gel strength [17]. These results were similar to those reported in Chen, Ma, Zhou, Liu, and Zhang [24], who showed that the pigskin gelatin extracted at 300 MPa for 15 min had a higher gel strength than other high-pressure parameters (100–500 MPa) and that the gel strength was not significantly different from 300 to 500 MPa.

#### 2.1.2. Solubility

Figure 1B shows that the solubility of the pressure-treated group is significantly higher than that of the control group (*p* < 0.05). As the pressure and duration increase, the solubility shows a trend consistent with the gel strength (Figure 1A); the results showed that the solubility of gelatin extracted at 300 MPa for 15 min was the highest, and its solubility was about 36% higher than that of gelatin under the same pressure for only 5 min. Numerous studies have found that other animal and plant proteins also show an upward and then downward trend under pressure treatment [20,25,26]. The strong impact and cavitation of high pressure may split highly-ordered protein macromolecules into smaller molecules, dissociating the quaternary structure of proteins and promoting protein–water interactions, leading to increased protein solubility [27]. Yang, Liu, Zeng, and Chen [15] found that high-pressure homogenization at 15 kpsi and 30 kpsi can respectively increase the solubility of fava bean protein by 98% and 99% compared with 0 kpsi, and they speculate that high pressure can transform large insoluble protein aggregates into soluble ones, thereby increasing protein solubility. Conversely, excessive pressure treatment can induce protein denaturation and form macromolecular aggregates, resulting in reduced protein solubility [20]. Liu, Zhang, Wang, Li, and Han [20] reported that when soybean protein isolate (SPI) glycated with flaxseed gum (FG) was incubated at 60 °C for 3 days at 0.1, 100, 200, and 300 MPa, the solubility of SPI-FG first increased and then decreased with increasing pressure, and the solubility was highest for proteins treated at 200 MPa (86.84%).

#### 2.1.3. Emulsifying Activity Index (EAI) and Emulsifying Stability Index (ESI)

As shown in Figure 1C,D, high pressure can significantly improve the emulsifying properties of cowhide gelatin (*p* < 0.05). Compared with the other treatments, the gelatin obtained at 300 MPa for 15 min showed the greatest EAI and ESI, after which both the EAI and ESI showed a downward trend as the pressure and duration continued to increase. Under appropriate pressure treatment, with increasing pressure and duration, non-covalent bonds (hydrophobic interactions and hydrogen bonds) that maintain the spatial structure of the protein are broken and the protein structure unfolds and expands with depolymerization, exposing more buried hydrophobic amino acids to the surface, thus increasing the surface activity and adsorption capacity of the protein at the oil–water interface, which leads to a significant increase in EAI [14,28]. Nonetheless, further increases in pressure and duration lead to the further unfolding of protein molecules and to the re-forming of molecular aggregates through hydrophobic interactions, disulfide bonds, electrostatic interactions, and hydrogen bonds, which results in a decrease in the protein surface area and molecular conformation flexibility and a decrease in EAI [29]. In contrast, Zhu, Lin, Ramaswamy, Yu, and Zhang [30] found that the EAI of rice bran proteins showed an upward trend with an increase in pressure (100–500 MPa), which may be due to the difference between plant raw materials and animal raw materials, resulting in different degrees of expansion, unfolding, or aggregation of different protein structures under high-pressure treatment.

The results for ESI from our experiment are similar to the research of Guan et al. [18] who reported the effect of soy protein isolate hydrolysate at different pressures (100–300 MPa) on the ESI of myofibrillar protein emulsions. Their results show that emulsions have the highest ESI when they are treated at 200 MPa for 4 h at 50 °C, indicating that pressure treatment enhances the ability of the oil–water interface membrane in the collagen emulsion to effectively resist external resistance. With the increase in pressure and duration, the structural unfolding and conformational changes of the protein cause the free sulfhydryl groups to be exposed to the aqueous medium, which strengthens the sulfhydryl–disulfide bond [31], resulting in an increase in the polymerization rate of collagen adsorbed at the oil–water interface of the emulsion that forms a strong viscoelastic film layer, thereby enhancing the stability of the emulsion system [18]. However, excessive pressure and duration cause undue protein denaturation, the flocculation of soluble aggregates, and a decrease in the viscoelasticity of the interfacial film, thereby deteriorating the mechanical properties of the interface and leading to a decrease in the stability of the emulsion [32].

#### 2.1.4. Foam Expansion (FE) and Foam Stability (FS)

As seen in Figure 1E,F, the FE and FS of the pressure-treated group were significantly higher than those of the control group and initially increased and then decreased (*p* < 0.05). The FS and FE of the gelatin extracted at 300 and 350 MPa for 15 min, respectively, were the highest. Our results are in line with a previous work on isolated pea protein under 0.1–600 MPa for 5 min at 23 °C, where the highest value of FE was obtained at 200 MPa, with the FE having reduced values as the pressure increased from 400 to 600 MPa [33]. It is reported that with an increase in pressure and duration, the droplet size of gelatin decreases, and the hydrophobicity of the protein surface increases, which causes protein molecules to adsorb at the air–water interface at a faster speed, thereby promoting bubble formation [34]. Chao, Jung, and Luko [33] conjecture that excessive pressure induced the unfolded protein to re-aggregate so that the conformational flexibility of the protein aggregates was low, and the ability to form a strong bubble encapsulation film at the air-water interface and form bubbles was significantly reduced. Diversely, Zhu, Lin, Ramaswamy, Yu, and Zhang [30] state that the FE of rice bran protein was significantly increased at 100–400 MPa for 10 min at 20 °C. This may be because high-pressure treatment damages the ionic and hydrogen bonds in animal protein and vegetable protein molecules to different degrees, resulting in a different ability to adsorb bubbles on the surface of the protein molecule.

Under a certain pressure treatment, an increase in pressure and duration significantly enhances the interactions between protein molecules in the membrane, and the aggregation of the protein turns the membrane thick and viscous, which prevents the foam from collapsing or breaking [30]. The optimum pressure (350 MPa) for the FS of gelatin treated for 5 min is higher than that for FS treated for other durations (at 300 MPa), which may be because the pressuring duration of 5 min is shorter compared with other high-pressure durations, resulting in an adsorption speed which did not reach the speed of collagen molecules on the air–water interface under 300 MPa. Furthermore, previous research reports that hydrogen bonds are extremely unstable in aqueous solutions, and thus excessive pressure and duration may cause the hydrogen bonds between polar proteins to be converted into protein–water interactions, thereby reducing foaming stability [34]. Interestingly, Qin et al. [35] found that the FS of walnut (*Juglans regia* L.) protein isolate treated for 20 min at high hydrostatic pressure at room temperature significantly increased as the pressure increased (300–600 MPa). The reason why our research is inconsistent with the results of Qin et al. [35] may be that the high temperature heating of the cowhide gelatin for a long time after pressure treatment strengthens the degree of spreading of the protein molecules, which then reconnect through non-covalent bonds to form larger molecular aggregates, resulting in a decrease in the stability of the water–air interface membrane.

### 2.2. Secondary Structure

**Figure 2:**
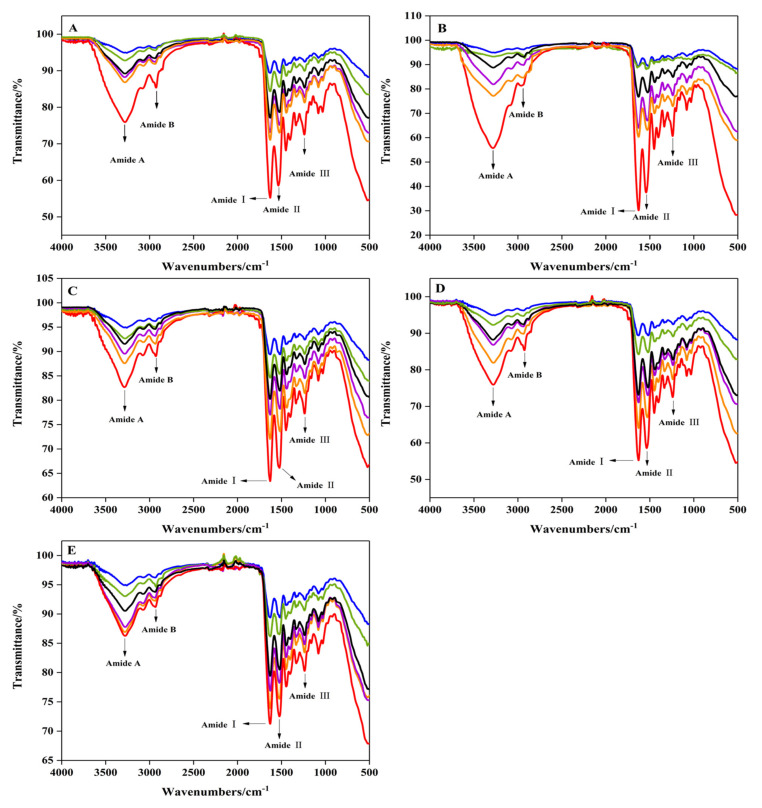
The effect of high hydrostatic pressure processing on the FTIR spectrum of cowhide collagen. High pressure 5 min (**A**); high pressure 10 min (**B**); high pressure 15 min (**C**); high pressure 20 min (**D**); high pressure 25 min (**E**). 0 MPa (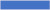); 200 MPa (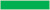); 250 MPa (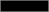); 300 MPa (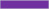); 350 MPa (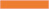); 400 MPa (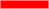). Amide A (amide A band in the FTIR spectrum); amide B (amide B band in the FTIR spectrum); amide I (amide I band in the FTIR spectrum); amide II (amide II band in the FTIR spectrum); amide III (amide III band in the FTIR spectrum).

Figure 2 shows that under different pressure treatments, the typical amide bands (amides A, B, I, II, III) of gelatin do exist in our spectrum. Compared with the control group, we observed a slight frequency shift in the amide A band (3200–3600 cm^−1^) in the pressure-treated group, suggesting high-pressure-induced changes in the interaction between collagen molecules. The peak for amide A under 200–350 MPa was red-shifted from 3381 cm^−1^ to a higher wave number of 3388 cm^−1^ compared with the control group, which might be caused by the strong mechanical force of high pressure that induces water molecules to embed inside the protein, causing the unfolding of collagen by breaking hydrogen bonds and hydrophobic protein–protein interactions [24,36]. However, when the pressure increased to 400 MPa, the peak for amide A moved back to a lower wave number of 3384 cm^−1^. This may be because under excessive pressure, the unfolding of the protein structure expands the polypeptide chain and promotes the binding of adjacent chains, causing the protein to re-aggregate, thereby enhancing the interaction between protein molecules [37]. This result is inconsistent with the study reported by Nan et al. [38], who found that the position of the amide A band of bullfrog skin collagen treated at 300 MPa for 15 min shifted slightly from 3426 cm^−1^ to 3408 cm^−1^ compared with non-pressure groups, indicating that more or stronger hydrogen bond structures may be formed in collagen molecules under such conditions. It is possible that the cowhide gelatin in our study was heat treated after the pressure treatment, which accelerated the destruction of hydrogen bonds and covalent bonds in the collagen molecules, causing the peak of the amide A band of the gelatin to move in the direction of a longer wavelength compared with the control group. Furthermore, as shown in Table 1, with the increase in duration, the α-helix contents of gelatin significantly reduced, with it first declining and then rising with the increase in pressure, whereas the opposite was true for random coils. This is in accordance with the results of Guo, Huang, Guo, Li, and Wang [32], who reported that the contents of random coils of kidney bean proteins first increase then decrease, while the α-helix decreases first then increases as the pressure increases (30–120 MPa). When the pressure and duration were increased to 400 MPa and 25 min, the degree of β-sheet formation was higher (77.36%), indicating that the protein interaction was enhanced and that the increase in β-sheet conformation may be due to the decrease in α-helix, β-turn, and random coils [37].

### 2.3. Intermolecular Force

#### 2.3.1. Hydrophobic Interactions

**Figure 3:**
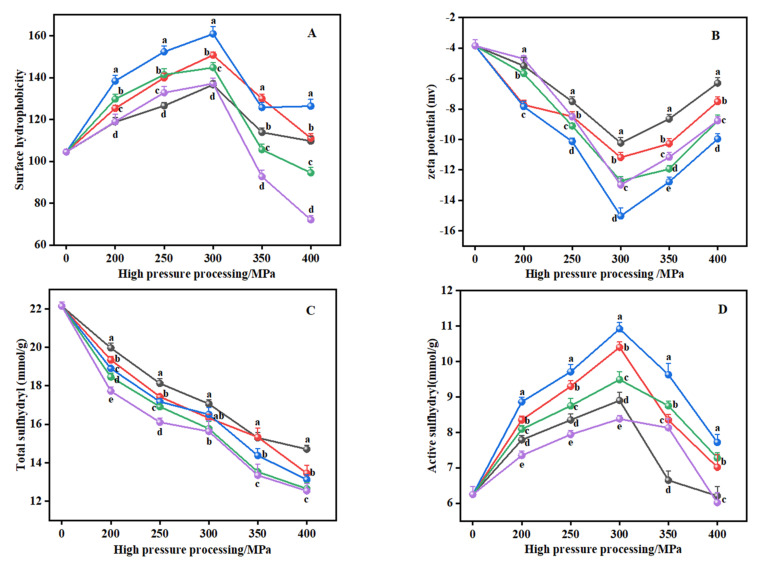
The effect of high hydrostatic pressure processing on the hydrophobic interactions (**A**), electrostatic interactions (**B**), and disulfide bonds contents (**C**,**D**) of cowhide collagen. The lowercase letters (a−e) indicate that different high−pressure durations have significant differences among the same pressure. High pressure 5 min (●); high pressure 10 min (●); high pressure 15 min (●); high pressure 20 min (●); high pressure 25 min (●).

Hydrophobic interactions between protein molecules are the main force that maintains the tertiary structure of proteins, and the surface hydrophobicity is mainly used to characterize the number of hydrophobic residues on the surface of protein molecules. The distribution of hydrophobic groups on a protein’s surface is closely related to the emulsification properties of the protein [39,40]. Figure 3A shows that under the same time duration, a lower pressure (200–300 MPa) can significantly improve the surface hydrophobicity of gelatin compared with the control group (*p* < 0.05), whereas a higher pressure (350–400 MPa) for more than 15 min has an inhibitory effect on surface hydrophobicity. This result is consistent with the law of emulsification of gelatin (Figure 1C,D). Other researchers have also observed similar trends in the surface hydrophobicity of sweet potato protein hydrolysates under high hydrostatic pressure (300–500 MPa) for 20 min at 25 °C, and their surface hydrophobicity was found to be highest at 400 MPa [21]. Based on the results of other experts on myofibril protein [41], proteins from scallops [42], soy protein isolates [22], and kidney beans [32] under high pressure, we conjecture that proper pressure treatment induces the denaturation and unfolding of protein molecules, promotes the entry of water molecules into the hydrophobic core of proteins, disrupts hydrophobic interactions within protein molecules, and exposes nonpolar amino acids, sulfhydryl groups, hydrophobic clusters, and hydrophobic groups previously buried in the internal regions of proteins and surrounded by a non-polar environment to the water environment. Here, more ANS can bind to the hydrophobic sites of the protein molecule, resulting in increased surface hydrophobicity. Moreover, other scientists have also studied the effect of high pressure on the surface hydrophobicity of myofibrillar protein from Oratosquilla oratoria muscles [43] and myosin-containing deacetylated konjac glucomannan [44]. Their results indicate that the surface hydrophobicity of animal-derived and plant-derived protein show a downward trend when pressure exceeds 300 MPa, which is more or less consistent with the results of this study. This could be explained by the fact that excessive pressure and duration causes the previously unfolded collagen molecule to re-agglomerate and fold, where the exposed hydrophobic groups are rapidly rearranged and embedded in this aggregate, resulting in a reduction in surface hydrophobicity.

#### 2.3.2. Disulfide Bonds

As shown in Figure 3C,D, high hydrostatic pressure significantly reduced the total sulfhydryl content of gelatin compared with the control group (*p* < 0.05), while the content of active sulfhydryl showed an increase at first and then a decrease. This result also supports our previous discussion that high pressure improves the functional properties of gelatin through the conversion of sulfhydryl–disulfide bonds in the protein structure. Studies report that most of the protein’s active sulfhydryl and free sulfhydryl is hidden inside the protein by Ellman’s reagent because of the sulfhydryl being located in the inaccessible region of the polypeptide chain [41]. On the other hand, high pressure may reduce the size of protein particles, resulting in the exposure of sulfhydryl groups inside the protein [22]. Therefore, the increase of active sulfhydryl suggests that high pressure may change the size and conformation of collagen and expose sulfhydryl to the protein surface [45,46]. However, Lv et al. [47] found that the active sulfhydryl content of myofibrillar protein from Tegillarca granosa were on a downward trend under pressure treatment (300 MPa for 5 min, 350 MPa for 3 or 5 min and 400 MPa for 1 min) at 20 °C. Similar to the discussion of surface hydrophobicity (Figure 3A), they conjecture that the protein aggregation process also intercalates the previously exposed active sulfhydryl, resulting in a decrease in the active sulfhydryl content, while its content may increase if the pressure is lower than 300 MPa. In addition, our result on total sulfhydryl content is in accordance with Cando, Herranz, Javier Borderias, and Moreno [48] who found that the total sulfhydryl content of Alaska Pollock surimi (Thera-grachalcogramma) decreases significantly as pressure increases (0, 150, 300 MPa) for 10 min at 10 °C. According to our results relating to surface hydrophobicity (Figure 3A), this may be because the high pressure breaks the hydrophobic interactions between protein molecules, shortening the cross-linking distance between sulfhydryl groups and promoting the formation of disulfide bonds [47].

#### 2.3.3. Electrostatic Interactions

Zeta potential is an important factor that reflects the interaction between charged protein particles, and represents the degree of stability of an emulsion system according to its electrostatic interaction [18]. As shown Figure 3B, the pressure-treated group had higher absolute values of zeta potential compared to the control samples (*p* < 0.05). Based on the results we observe in FTIR (Figure 2, Table 1), this is likely due to the random rupture and unfolding of collagen under high pressure exposing the charged residues of amino acids on the protein surface and increasing the charged groups on the protein surface [22]. Under the same duration, the absolute value of zeta potential first increases and then decrease as the pressure increases, reaching its maximum at 300 MPa (15.03 mV). At the same pressure, the highest absolute value is reached with a duration of treatment of 15 min, indicating that the state of particles in the collagen structure tends to be the most stable at 300 MPa for 15 min, as corroborated by the ESI (Figure 1D). This may be attributed to the decomposition of proteins into small particles with the increase in pressure and duration, resulting in a significant increase in the surface area of protein molecules. Meanwhile, the charged residues of amino acids continue to be exposed, and the newly exposed groups interact with water molecules by combining with hydrogen bonds, which leads to an increase in the electrostatic repulsion between the same charges, delays the formation of protein aggregates, and improves the solubility of proteins in water [14,37]. Similar observations and trends were reported in our solubility results (Figure 1B). Although the absolute value of zeta potential of gelatin under 300 MPa for 15 min is the greatest (15.03 mV), the absolute range of it that can maintain the stability of protein solution is 20 to 30 mV [18,27], which shows that the electrostatic repulsion between charges in gelatin solution is still not very strong under this pressure condition. Therefore, in conjunction with our ESI results (Figure 1D), we hypothesize that this is due to the high viscoelastic film formed by the adsorption of collagen and peptides at the oil–water interface, which enhances the electrostatic repulsion or spatial site resistance between the protein particles and thus increases the stability of the gelatin solution. Furthermore, our results relating to the decrease in the absolute value of zeta potential are consistent with the findings of Chen, Zhou, Xu, Zhou, and Liu [37] who find that the absolute value of the zeta potential of freeze-dried myofibrillar proteins powder at 20,000 psi was significantly lower than the absolute value at 15,000 psi. They consider that this may be due to disulfide bond cross-linking or hydrophobic interactions causing a small amount of protein to aggregate under excessive pressure, reducing the net charge content of the protein surface and the polarity of the protein and resulting in a reduction in the absolute value of zeta potential.

#### 2.3.4. Hydrogen Bonds

Tyrosine residues are an important group for forming hydrogen bonds. Based on this, we used Raman spectroscopy to detect the hydrogen bonds of cowhide gelatin. The values of I850/I830 determines whether the phenolic hydroxyl group of the tyrosine residue is exposed or buried [49]. Compared to the unpressurised group (0.9241), the I850/I830 of gelatin at 200–350 MPa ranged from 0.9443 to 1.0132 and was less than 0.9 for I850/I830 at 400 MPa (Table 2). This suggests that under the appropriate pressure treatment, the tyrosine residues of the collagen molecule are predominantly exposed and are able to combine with water molecules to form more hydrogen bonds. However, I855/I830 is significantly reduced at higher pressures (400 MPa), in conjunction with the results relating to hydrophobic interactions (Figure 3A), possibly due to the high-pressure effect causing the phenolic hydroxyl groups of the exposed tyrosine residues and the hydrophobic groups to become embedded in the protein molecule during protein aggregation, leading to a reduction in Raman intensity [50]. Moreover, it can be seen from Table 2 that the Nexposed show a trend of first increasing and then decreasing as the pressure and duration increase, and the highest Nexposed (0.6842) was obtained at 300 MPa for 15 min. Also, Zhang, Yang, Tang, Chen, and You [51] report that the tyrosine phenolic hydroxyl group of myofibrillar protein gel exposed to a water environment increases significantly as pressure increases (0.1–200 MPa), while its content conspicuously decreases when the pressure continues to increase (300–500 MPa). They believe that under a pressure of 0.1–200 MPa, more tyrosine phenolic hydroxyl groups are exposed to the aqueous environment and form hydrogen bonds with water molecules, while these groups remain hidden in the hydrophobic microenvironment and thereby form more hydrogen bonds with protein molecules at 300–500 MPa. The same trend was found in our research, suggesting that protein–protein hydrogen bonding increased and protein–water hydrogen bonding decreased when the pressure and duration continued to increase (>300 MPa, >15 min), which is also consistent with the changes in the α-helix we found in the FTIR spectrum of cowhide gelatin (Table 1).

### 2.4. Principal Component Analysis (PCA)

**Figure 4:**
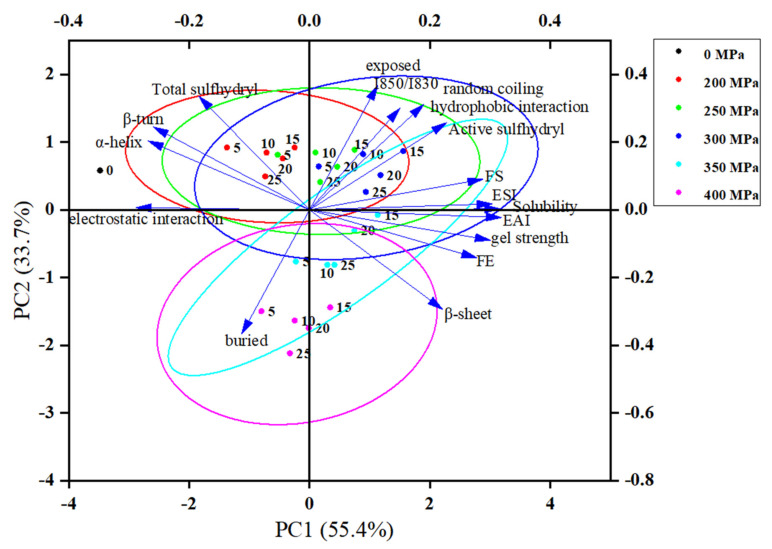
Factor loading diagram of two principal components of the functional properties and structural characteristics of cowhide gelatin induced by high hydrostatic pressure.

Two principal component factors of the functional properties and structural characteristics of cowhide gelatin induced by high hydrostatic pressure were extracted by PCA. The cumulative contribution rate of the two principal components was 89.113%, of which the contribution rate of the first principal component (PC1) was 55.434%, and the contribution rate of the second principal component (PC2) was 33.679%. PC1 mainly distinguished different pressure groups (0 MPa, 200 MPa, 250 MPa, 300 MPa, 350 MPa, and 400 MPa) (Figure 4). The gelatins in the control group (0 MPa) were in the negative quadrant of PC1, and they were clustered with total sulfhydryl, electrostatic interactions, α-helix, and β-turn. This phenomenon indicated that the total sulfhydryl, electrostatic interaction, α-helix, and β-turn contents of cowhide gelatin at 0 MPa were significantly higher than those of other pressure groups. This result is also consistent with the results in Table 1, Figure 3B,C in this study. As the pressure increased, the 200 MPa group, 250 MPa group, and 300 MPa group moved sequentially from the negative quadrant of PC1 to its positive quadrant. However, the 350 MPa group and the 400 MPa group sequentially moved towards the negative quadrant when the pressure continued to increase. The 300 MPa group was densely distributed with Nexposed, I850/I830, random coiling, hydrophobic interaction, active sulfhydryl, FS, ESI, solubility, EAI, and gel strength, indicating that the contents of these indicators first increased and then decreased with increasing pressure, with the highest at 300 MPa. These results are in line with those of Table 1 and Table 2, Figure 1 and Figure 3A,D. PC2 mainly distinguished different duration groups (0 min, 5 min, 10 min, 15 min, 20 min, and 25 min). As can be seen from Figure 4, apart from the Nburied and β-sheet, the best duration for which was 25 min, the best duration for the other indexes was 15 min. These results are also in keeping with the research results of this paper.

## 3. Conclusions

High hydrostatic pressure treatment with different pressures and durations can change the structure of cowhide collagen to varying degrees, and the promotion effect of structure unfolding first increases and then decreases with increases in pressure and duration. In particular, high pressure at 300 MPa for 15 min can maximize the expansion and unfolding of the collagen structure, break the hydrogen bonds that maintain the secondary structure, and expose more hydrophobic groups and amino acid residues. Moreover, the gelatin obtained by 300 MPa (15 min) had the highest gel strength and the best functional properties. Therefore, appropriate pressure treatment can promote the unfolding of and change the structure of cowhide collagen, thus improving the functional properties and utilization value of the cowhide. Importantly, this study provides a theoretical basis and technical guidance for the application of high hydrostatic pressure in cowhide and thusly for reductions in both waste from cowhide byproducts and environmental contamination. In future research, we hope to further explore the applications of high hydrostatic pressure-treated cowhide in the food industry.

## 4. Materials and Methods

### 4.1. Materials

The cowhides were provided by Jingxing Halal Meat Co., Ltd. (Lanzhou, China). The removal of cowhide hair was performed according to the method of He, Gao, Han, Yu, and Zang [52], and 95% of the hair was removed. The cowhide was also cleaned, excess fat and muscle were removed, and the hide was cut into small pieces (1 × 1 cm^2^). After drying the cowhide at 105 °C for 2 h, it was degreased by Soxhlet extraction. Subsequently, five times the volume (*w*/*v*) of NaCl solution (1%) was added to the cowhide, and the cowhide was soaked for 12 h to remove non-collagen substances and later washed three times with distilled water. After the cowhide was mixed with distilled water (the ratio of material to liquid was 1:1.5), it was placed into a polyethylene bag for vacuum packaging. Pure water was used as the medium, and then the packaged cowhide solution was pressurized at 200, 250, 300, 350, and 400 MPa for 5, 10, 15, 20, and 25 min using a high-pressure vessel supplied by Baotou Kefa High Voltage Technology Co., Ltd. (Baotou, China) while a cooling device was used to keep the temperature at 25 ± 2 °C. Unpressurized and post-pressurized cowhide were heated at 105 °C for 6 h, with the hair and other impurities being repeatedly filtered, was cooled until the gel system was stable, and then freeze-dried [24,38]. Chemical reagents were all of analytical grade.

### 4.2. Functional Characteristics

#### 4.2.1. Gel Strength and Solubility

The gelatin solution with a mass fraction of 6.67% were completely dissolved in a 60 °C water bath then cooled at 4 °C for 16 h. The gel strength was then measured by a TA-XT2 Express texture analyzer (Beijing, China) prepared with a load cell (5 kg), a cross-head speed (1 mm/s), and a flat-faced cylindrical plunger (12.7 mm diameter). Gel strength was expressed as the maximum force (g) of the plunger into the gel at a depth of 4 mm. The solubility of cowhide gelatin was measured following the testing method described by Kuan, Nafchi, Huda, Ariffin, and Karim [53]. The gelatin solution (1%, *w*/*v*) was heated at a constant temperature of 60 °C for 15 min, and the pH value was adjusted to the range of 2 to 11. The gelatin solution was then centrifuged at 3000× *g* for 10 min at 4 °C, and the concentration of supernatant was determined by the biuret method at a wavelength of 280 nm in a UV-spectrophotometer (Shanghai, China) [53]. The gelatin solubility was calculated as follows:

Gelatin solubility (%) = Supernatant concentration/Gelatin solution concentration × 100

#### 4.2.2. Emulsifying and Foaming Properties

The Emulsifying activity index and emulsifying stability index were determined based on the method of Xu et al. [54]. Peanut oil (2 mL) and 6 mL of 3% (*w*/*v*) gelatin solution were mixed and homogenized at a high speed of 10,000 r/min for 1 min. Then, the homogenized emulsion was diluted 100 times by adding 0.1% SDS at 0 min and 10 min. The absorbance values at both time points were measured at 500 nm. The foam expansion and foam stability of cowhide gelatin were measured as described by Xu et al. [54]. The gelatin solution (3%, *w*/*v*) was homogenized at 12,000 rpm for 1 min, and then allowed to stand at room temperature for 0 min and 30 min. The FE and FS were calculated according to the following formulas:

FE (%) = (Total volumes after homogenization/original volumes before homogenization)/original volumes before homogenization × 100

FS (%) = (Total volumes after standing for 30 min/original volumes before homogenization)/original volumes before homogenization × 100

### 4.3. Secondary Structure

The FTIR spectroscopy of gelatin was determined using a FTIR infrared spectrometer (Nicolet iS20, California Analytical Instruments Co., Ltd., Orange, CA, USA) according to the method of He, Gao, Han, Yu, and Zang [52]. After the freeze-dried cowhide gelatin was mixed with KBr (1:20) and compressed into tablets, we measured the compressed mixture with a resolution of 4 cm^−1^ and a spectral range of 500–4000 cm^−1^ using the KBr as the scanning background. The obtained spectrum was curve-fitted and analyzed by Peakfit 4.12.

### 4.4. Intermolecular Force

#### 4.4.1. Hydrophobic Interactions (Surface Hydrophobicity) and Disulfide Bonds (Total Sulfhydryl Groups and Active Sulfhydryl)

The surface hydrophobicity of gelatin was determined using a fluorescence probe (ANS, 8 mmoL L^−1^) according to the method depicted by Nan et al. [38]. The gelatin solution was diluted to 0.1%, 0.3%, 0.5%, 0.7%, 0.9% by 0.01 moL L^−1^ phosphate buffer (pH 6.1) containing 0.15 moL L^−1^ NaCl. Diluent (4 mL) was mixed with 80 µL of 8 mmoL L^−1^ ANS containing 0.01 moL L^−1^ phosphate buffer (pH 6.1). The fluorescence intensity (excitation wavelength 390 nm, emission wavelength 470 nm) was measured by a F-2500 spectrofluorophotometer (Hitachi, Tianjin, China), and the surface hydrophobicity was expressed as the slope. The content of total sulfhydryl groups and active sulfhydryl content in the cowhide gelatin were determined according to Ellman et al. [55]. The absorbance of the gelatin solution was measured at 412 nm using a UV-spectrophotometer (Shanghai, China). Results were expressed as millimoles of sulfhydryl groups per gram of gelatin.

#### 4.4.2. Electrostatic Interactions (Zeta Potentials) and Hydrogen Bonds (Raman Spectra)

The zeta potentials of gelatin (1 mg mL^−1^) were measured at room temperature (25 °C) using a Zeta Nano Analyzer supplied by Brookhaven Instruments CO., Ltd. (Brookhaven, GA, USA) according to the method of Guan et al. [18]. Raman spectra of the gelatin were measured using a High Speed and High Resolution Confocal Microscope Raman Spectrometer provided by HORIBA Scientific Instruments CO., Ltd. (LabRAM Odyssey, Paris, France) at an excitation wavelength of 532 nm at room temperature (25 °C) [50].

### 4.5. Statistical Analysis

To ensure the robustness of our results, we repeated the entire experiment independently four times. We displayed our data as means and standard deviations (mean ± SD). One-way analysis of variance (ANOVA) was used to determine the significance of the main effect, and the significant differences (*p* < 0.05) among different groups were identified using Duncan’s multiple range test. All statistical analysis was performed using SPSS 20.0 data analysis software. All data were analyzed using SPSS 20.0 with KMO test coefficient >0.5 and Bartlett’s sphericity test *p*-value < 0.05, followed by principal component analysis (PCA) using Origin (version 2021).

## Figures and Tables

**Table 1:** Effect of high hydrostatic pressure on the secondary structure content evaluated from deconvoluted FTIR spectra of cowhide collagen.

P (MPa)	T (min)					S.E	Sig.		
	5	10	15	20	25		P	T	I
α-helix (%)									
0	13.52 ^Aa^ ± 0.24	13.52 ^Aa^ ± 0.24	13.52 ^Aa^ ± 0.24	13.52 ^Aa^ ± 0.24	13.52 ^Aa^ ± 0.24	<0.01	**	**	**
200	10.27 ^Ba^ ± 0.18	9.79 ^Bb^ ± 0.23	9.38 ^Bbc^ ± 0.43	9.11 ^Bc^ ± 0.21	8.69 ^Bd^ ± 0.19				
250	9.53 ^Ca^ ± 0.19	8.95 ^Cb^ ± 0.14	8.23 ^Cc^ ± 0.23	7.93 ^Cc^ ± 0.25	7.63 ^Cd^ ± 0.31				
300	8.38 ^Da^ ± 0.11	7.87 ^Db^ ± 0.21	7.42 ^Dbc^ ± 0.24	7.12 ^Dc^ ± 0.21	6.94 ^Dc^ ± 0.29				
350	6.84 ^Fa^ ± 0.33	6.75 ^Ea^ ± 0.19	6.21 ^Eb^ ± 0.29	6.27 ^Eb^ ± 0.15	6.22 ^Eb^ ± 0.31				
400	7.89 ^Ea^ ± 0.22	7.61 ^Dab^ ± 0.32	7.12 ^Db^ ± 0.15	7.35 ^Db^ ± 0.17	7.13 ^Db^ ± 0.26				
β-sheet (%)									
0	58.38 ^Ea^ ± 1.32	58.38 ^Ea^ ± 1.32	58.38 ^Ea^ ± 1.32	58.38 ^Ea^ ± 1.32	58.38 ^Ea^ ± 1.32	<0.01	**	**	**
200	65.29 ^De^ ± 0.27	66.97 ^Dd^ ± 0.35	67.93 ^Dc^ ± 0.23	68.39 ^Db^ ± 0.22	69.21 ^Da^ ± 0.43				
250	67.31 ^Ce^ ± 0.35	68.99 ^Cd^ ± 0.31	70.24 ^Cc^ ± 0.45	71.13 ^Cb^ ± 0.28	71.99 ^Ca^ ± 0.31				
300	70.27 ^Bd^ ± 0.41	71.31 ^Bc^ ± 0.39	72.25 ^Bb^ ± 0.52	73.23 ^Ba^ ± 0.41	74.14 ^Ba^ ± 0.56				
350	74.17 ^Ac^ ± 0.39	76.26 ^Ab^ ± 0.48	76.17 ^Ab^ ± 0.44	76.52 ^Ab^ ± 0.34	77.52 ^Aa^ ± 0.39				
400	74.19 ^Ad^ ± 0.49	75.37 ^Ac^ ± 0.41	75.91 ^Abc^ ± 0.36	76.21 ^Ab^ ± 0.38	77.36 ^Aa^ ± 0.45				
β-turn (%)									
0	16.48 ^Aa^ ± 0.23	16.48 ^Aa^ ± 0.23	16.48 ^Aa^ ± 0.23	16.48 ^Aa^ ± 0.23	16.48 ^Aa^ ± 0.23	<0.01	**	*	**
200	11.69 ^Ba^ ± 0.52	10.38 ^Bb^ ± 0.58	9.87 ^Bb^ ± 0.72	9.32 ^Bb^ ± 0.49	9.03 ^Bb^ ± 0.68				
250	9.82 ^Ca^ ± 0.21	8.21 ^Cb^ ± 0.29	7.56 ^Cc^ ± 0.35	7.21 ^Cc^ ± 0.42	7.09 ^Cc^ ± 0.54				
300	7.79 ^Da^ ± 0.19	6.58 ^Db^ ± 0.22	5.94 ^Dc^ ± 0.32	5.25 ^Dd^ ± 0.33	5.11 ^Dd^ ± 0.32				
350	6.83 ^Ea^ ± 0.14	5.23 ^Eb^ ± 0.16	4.61 ^Ec^ ± 0.26	4.42 ^Ec^ ± 0.32	4.02 ^Ed^ ± 0.33				
400	6.79 ^Ea^ ± 0.12	5.33 ^Eb^ ± 0.15	4.63 ^Ec^ ± 0.23	4.45 ^Ec^ ± 0.27	4.03 ^Ed^ ± 0.12				
random coiling (%)									
0	11.62 ^Da^ ± 0.27	11.62 ^Ca^ ± 0.27	11.62 ^Ea^ ± 0.27	11.62 ^Da^ ± 0.27	11.62 ^Ca^ ± 0.27	<0.01	**	n.s	*
200	12.75 ^Cb^ ± 0.19	12.86 ^Bb^ ± 0.14	12.82 ^Cb^ ± 0.12	13.18 ^Ca^ ± 0.17	13.07 ^Bab^ ± 0.15				
250	13.34 ^Bc^ ± 0.11	13.85 ^Aa^ ± 0.31	13.97 ^Ba^ ± 0.16	13.73 ^Ba^ ± 0.11	13.69 ^Ab^ ± 0.11				
300	13.56 ^Ab^ ± 0.14	14.24 ^Aa^ ± 0.18	14.29 ^Aa^ ± 0.15	14.4 ^Aa^ ± 0.14	13.81 ^Ab^ ± 0.13				
350	12.16 ^Cc^ ± 0.21	11.76 ^Cd^ ± 0.12	13.01 ^Ca^ ± 0.13	13.09 ^Ca^ ± 0.12	12.74 ^Bb^ ± 0.22				
400	11.13 ^Ed^ ± 0.12	11.69 ^Cc^ ± 0.14	12.34 ^Da^ ± 0.12	11.99 ^Db^ ± 0.13	11.48 ^Cc^ ± 0.14				

The lowercase letters indicate that different high-pressure durations have significant differences. The capital letters indicate that different pressures have significant differences. S.E: Standard error; Sig.: significance; n.s: not significant; P: pressure; T: time; I: interaction. * *p* < 0.05; ** *p* < 0.01.

**Table 2:** The conjugate bimodal ratio of different high hydrostatic pressure treatments to cowhide collagen I850/I830 double band and the mole fraction of exposed (hidden) tyrosine residues.

P (MPa)	T (min)					S.E	Sig.		
	5	10	15	20	25		P	T	I
I850/I830									
0	0.9241 ^Ea^ ± 0.0012	0.9241 ^Ea^ ± 0.0012	0.9241 ^Ea^ ± 0.0012	0.9241 ^Ca^ ± 0.0012	0.9241 ^Da^ ± 0.0012	<0.01	**	**	**
200	1.0030 ^Cd^ ± 0.0001	1.0040 ^Cc^ ± 0.0008	1.0078 ^Da^ ± 0.0009	1.0056 ^Bb^ ± 0.0002	1.0023 ^Be^ ± 0.0021				
250	1.0064 ^Bb^ ± 0.0008	1.0077 ^Ba^ ± 0.0003	1.0090 ^Ca^ ± 0.0014	1.0062 ^Ab^ ± 0.0001	1.0045 ^Bc^ ± 0.0007				
300	1.0096 ^Ac^ ± 0.0003	1.0119 ^Ab^ ± 0.0006	1.0132 ^Aa^ ± 0.0003	1.0061 ^Ae^ ± 0.0002	1.0075 ^Ad^ ± 0.0011				
350	0.9495 ^Dd^ ± 0.0005	0.9443 ^Dd^ ± 0.0013	1.0091 ^Ba^ ± 0.0022	1.0058 ^ABb^ ± 0.0009	0.9782 ^Cc^ ± 0.0014				
400	0.8902 ^Fab^ ± 0.0007	0.8823 ^Fd^ ± 0.0004	0.8911 ^Fa^ ± 0.0003	0.8901 ^Db^ ± 0.0003	0.8891 ^Ec^ ± 0.0006				
Nexposed									
0	0.5655 ^Ea^ ± 0.0002	0.5655 ^Ea^ ± 0.0002	0.5655 ^Da^ ± 0.0002	0.5655 ^Ca^ ± 0.0002	0.5655 ^Ea^ ± 0.0002	<0.01	**	**	**
200	0.6707 ^Cd^ ± 0.0003	0.6721 ^Cc^ ± 0.0003	0.6771 ^Ca^ ± 0.0004	0.6741 ^Bb^ ± 0.0003	0.6697 ^Ce^ ± 0.0005				
250	0.6752 ^Bc^ ± 0.0001	0.6770 ^Bb^ ± 0.0001	0.6787 ^Ba^ ± 0.0003	0.6750 ^Ac^ ± 0.0001	0.6726 ^Bd^ ± 0.0003				
300	0.6795 ^Ac^ ± 0.0002	0.6826 ^Ab^ ± 0.0004	0.6842 ^Aa^ ± 0.0001	0.6748 ^Ae^ ± 0.0004	0.6767 ^Ad^ ± 0.0004				
350	0.5993 ^Dd^ ± 0.0004	0.5924 ^De^ ± 0.0011	0.6788 ^Ba^ ± 0.0002	0.6744 ^Bb^ ± 0.0002	0.6376 ^Dc^ ± 0.0002				
400	0.5203 ^Fb^ ± 0.0003	0.5097 ^Fd^ ± 0.0005	0.5215 ^Ea^ ± 0.0005	0.5201 ^Db^ ± 0.0005	0.5188 ^Fc^ ± 0.0004				
Nburied									
0	0.4345 ^Ba^ ± 0.0004	0.4345 ^Ba^ ± 0.0004	0.4345 ^Ba^ ± 0.0004	0.4345 ^Ba^ ± 0.0004	0.4345 ^Ba^ ± 0.0004	<0.01	**	**	**
200	0.3293 ^Db^ ± 0.0003	0.3279 ^Dc^ ± 0.0002	0.3229 ^De^ ± 0.0005	0.3259 ^Cd^ ± 0.0003	0.3303 ^Da^ ± 0.0005				
250	0.3248 ^Eb^ ± 0.0003	0.3230 ^Ec^ ± 0.0004	0.3213 ^Ed^ ± 0.0005	0.3250 ^Db^ ± 0.0002	0.3274 ^Ea^ ± 0.0003				
300	0.3205 ^Fc^ ± 0.0006	0.3174 ^Fd^ ± 0.0003	0.3158 ^Ce^ ± 0.0006	0.3252 ^Da^ ± 0.0001	0.3233 ^Fb^ ± 0.0004				
350	0.4007 ^Cb^ ± 0.0008	0.4076 ^Ca^ ± 0.0002	0.3212 ^Ee^ ± 0.0002	0.3256 ^Cd^ ± 0.0002	0.3624 ^Cc^ ± 0.0003				
400	0.4797 ^Ac^ ± 0.0005	0.4903 ^Aa^ ± 0.0003	0.4785 ^Ad^ ± 0.0003	0.4799 ^Ac^ ± 0.0003	0.4812 ^Ab^ ± 0.0002				

The lowercase letters indicate that different high-pressure durations have significant differences. The capital letters indicate that different pressures have significant differences. S.E: Standard error; Sig.: significance; P: pressure; T: time; I: interaction. ** *p* < 0.01.

## Data Availability

The data generated from the study is clearly presented and discussed in the manuscript.

## References

[1] Toldra F., Mora L., Reig M. (2016). New insights into meat by-product utilization. Meat Sci..

[2] Jayathilakan K., Sultana K., Radhakrishna K., Bawa A.S. (2012). Utilization of byproducts and waste materials from meat, poultry and fish processing industries: A review. J. Food Sci. Technol..

[3] Shen X., Zhang M., Bhandari B., Gao Z. (2018). Novel technologies in utilization of byproducts of animal food processing: A review. Crit. Rev. Food Sci. Nutr..

[4] Ahmad T., Ismail A., Ahmad S.A., Khalil K.A., Kee L.T., Awad E.A., Sazili A.Q. (2019). Physicochemical characteristics and molecular structures of gelatin extracted from bovine skin: Effects of actinidin and papain enzymes pretreatment. Int. J. Food Prop..

[5] Shahbandeh M. (2017). Distribution Share of the Gelatin Market Worldwide in 2016, by Type of Raw Material. https://www.statista.com/statistics/712112/distribution-gelatin-market-worldwide/.

[6] Nitsuwat S., Zhang P., Ng K., Fang Z. (2021). Fish gelatin as an alternative to mammalian gelatin for food industry: A meta-analysis. LWT-Food Sci. Technol..

[7] Boran G., Pancar E.D., Andic S. (2016). Comparative effects of fish and cow gelatins and locust bean gum on chemical, textural, and sensory properties of yogurt. J. Aquat. Food Prod. Technol..

[8] Nuez S.M., Cárdenas C., Pinto M., Valencia P., Almonacid S. (2020). Bovine skin gelatin hydrolysates as potential substitutes for polyphosphates: The role of degree of hydrolysis and pH on water holding capacity. J. Food Sci..

[9] Ma Y.S., Zhao H.J., Zhao X.H. (2019). Comparison of the effects of the alcalase-hydrolysates of caseinate, and of fish and bovine gelatins on the acidification and textural features of set-style skimmed yogurt-type products. Foods.

[10] Ahmad T., Ismail A., Ahmad S.A., Khalil K.A., Kumar Y., Adeyemi K.D., Sazili A.Q. (2017). Recent advances on the role of process variables affecting gelatin yield and characteristics with special reference to enzymatic extraction: A review. Food Hydrocoll..

[11] Noor N.Q.I.M., Razali R.S., Ismail N.K., Ramli R.A., Razali U.H.M., Bahauddin A.R., Zaharudin N., Rozzamri A., Bakar J., Shaarani S.M. (2021). Application of Green Technology in Gelatin Extraction: A Review. Processes.

[12] Park J.H., Choe J.H., Kim H.W., Hwang K.E., Song D.H., Yeo E.J., Kim H.Y., Choi Y.S., Lee S.H., Kim C.J. (2013). Effects of various extraction methods on quality characteristics of duck feet gelatin. Korean J. Food Sci. Anim. Resour..

[13] Tomas B., Vibeke O., Anita S., Kemal A., Kathrine H.B., Claire G., Anna-Sophie S., Marie D.L., Christian H., Dagmar A.B. (2020). High-pressure processing of meat: Molecular impacts and industrial applications. Compr. Rev. Food Sci. Food Saf..

[14] Bai Y., Zeng X., Zhang C., Zhang T., Xu X. (2021). Effects of high hydrostatic pressure treatment on the emulsifying behavior of myosin and its underlying mechanism. LWT-Food Sci. Technol..

[15] Yang J., Liu G., Zeng H., Chen L. (2018). Effects of high pressure homogenization on faba bean protein aggregation in relation to solubility and interfacial properties. Food Hydrocoll..

[16] Xing S.M., Jian R.L., Chen J.R., Shu M.Y., Yong M.Y. (2015). Changes in gel properties and water properties of nemipterus virgatus surimi gel induced by high-pressure processing. LWT-Food Sci. Technol..

[17] Guo Z., Li Z., Wang J., Zheng B. (2019). Gelation properties and thermal gelling mechanism of golden threadfin bream myosin containing cacl2 induced by high pressure processing. Food Hydrocoll..

[18] Guan H.N., Diao X.Q., Liu D.Y., Han J.C., Kong B.H., Liu D.Y., Gao C.Z., Zhang L.L. (2020). Effect of high-pressure processing enzymatic hydrolysates of soy protein isolate on the emulsifying and oxidative stability of myofibrillar protein-prepared oil-in-water emulsions. J. Sci. Food Agric..

[19] Zhang Y., Ren Y., Bi Y., Wang Q., Chen F. (2019). Review: Seafood allergy and potential application of high hydrostatic pressure to reduce seafood allergenicity. Int. J. Food Eng..

[20] Liu D., Zhang L., Wang Y., Li Z., Han J. (2020). Effect of high hydrostatic pressure on solubility and conformation changes of soybean protein isolate glycated with flaxseed gum. Food Chem..

[21] Falade E.O., Mu T.H., Zhang M. (2021). Improvement of ultrasound microwave-assisted enzymatic production and high hydrostatic pressure on emulsifying, rheological and interfacial characteristics of sweet potato protein hydrolysates. Food Hydrocoll..

[22] Tan M., Xu J., Gao H., Yu Z., Zheng Z. (2021). Effects of combined high hydrostatic pressure and ph-shifting pretreatment on the structure and emulsifying properties of soy protein isolates. J. Food Eng..

[23] Sezer P., Okur I., Oztop M.H., Alpas H. (2019). Improving the physical properties of fish gelatin by high hydrostatic pressure (hhp) and ultrasonication (us). Int. J. Food Sci. Technol..

[24] Chen L.Q., Ma L., Zhou M.R., Liu Y., Zhang Y.H. (2014). Effects of pressure on gelatinization of collagen and properties of extracted gelatins. Food Hydrocoll..

[25] Zheng H., Han M., Yun B., Xu X., Zhou G. (2018). Combination of high pressure and heat on the gelation of chicken myofibrillar proteins. Innov. Food Sci. Emerg. Technol..

[26] Xue S., Wang C., Yuan H., Bian G., Zhou G. (2019). Application of high-pressure treatment improves the in vitro protein digestibility of gel-based meat product. Food Chem..

[27] Chen X., Xu X., Zhou G. (2016). Potential of high pressure homogenization to solubilize chicken breast myofibrillar proteins in water. Innov. Food Sci. Emerg. Technol..

[28] Chen X., Tume R.K., Xiong Y., Xu X.L., Nishiumi T. (2017). Structural modification of myofibrillar proteins by high-pressure processing for functionally improved, value-added and healthy muscle gelled foods. Crit. Rev. Food Sci. Nutr..

[29] Ya L.F., Oey I., Bremer P., Carne A., Silcock P. (2019). Modifying the functional properties of egg proteins using novel processing techniques: A review. Compr. Rev. Food Sci. Food Saf..

[30] Zhu S.M., Lin S.L., Ramaswamy H.S., Yu Y.Q., Zhang T. (2017). Enhancement of functional properties of rice bran proteins by high pressure treatment and their correlation with surface hydrophobicity. Food Bioprocess Technol..

[31] Taghi G.S.M., Hernández-Ortega C., Jorge W.C., Predrag P., Barba F.J., Kumar M., Escobedo-Avellaneda Z., Roohinejad S. (2018). High pressure processing of food-grade emulsion systems: Antimicrobial activity, and effect on the physicochemical properties. Food Hydrocoll..

[32] Guo Z.W., Huang Z.X., Guo Y.N., Li B.L., Wang Z.J. (2021). Effects of high-pressure homogenization on structural and emulsifying properties of thermally soluble aggregated kidney bean (*Phaseolus vulgaris* L.) proteins. Food Hydrocoll..

[33] Chao D., Jung S., Luko A.R. (2017). Physicochemical and functional properties of high pressure-treated isolated pea protein. Innov. Food Sci. Emerg. Technol..

[34] Baier A.K., Knorr D. (2015). Influence of high isostatic pressure on structural and functional characteristics of potato protein. Food Res. Int..

[35] Qin Z.H., Guo X.F., Lin Y., Chen J.L., Liao X.J., Hu X.S., Wu J.H. (2013). Effects of high hydrostatic pressure on physicochemical and functional properties of walnut (*Juglans regia* L.) protein isolate. J. Sci. Food Agric..

[36] Chen X., Xu X., Han M., Zhou G., Chen C., Li P. (2016). Conformational changes induced by high-pressure homogenization inhibit myosin filament formation in low ionic strength solutions. Food Res. Int..

[37] Chen X., Zhou R., Xu X., Zhou G., Liu D. (2017). Structural modification by high-pressure homogenization for improved functional properties of freeze-dried myofibrillar proteins powder. Food Res. Int..

[38] Nan J., Zou M.L., Wang H.B., Xu C.Z., Zhang J.T., Wei B.M., He L., Xu Y.L. (2018). Effect of ultra-high pressure on molecular structure and properties of bullfrog skin collagen. Int. J. Biol. Macromol..

[39] Kang D.C., Zou Y.H., Cheng Y.P., Xing L.J., Zhou G.H., Zhang W.G. (2016). Effects of power ultrasound on oxidation and structure of beef proteins during curing processing. Ultrason. Sonochem..

[40] Jiang S., Zhao N.Y., Wu J., Li C. (2021). Ultrasonic treatment increased functional properties and in vitro digestion of actomyosin complex during meat storage. Food Chem..

[41] Zhang Z., Yang Y., Zhou P., Zhang X., Wang J. (2017). Effects of high pressure modification on conformation and gelation properties of myofibrillar protein. Food Chem..

[42] Wu D., Wu C., Wang Z.Y., Fan F.J., Chen H., Ma W.C., Du M. (2019). Effects of high pressure homogenize treatment on the physicochemical and emulsifying properties of proteins from scallop (*Chlamys farreri*). Food Hydrocoll..

[43] Li G.S., Chen Y.T., Xuan S.F., Lv M.C., Zhang J.J., Lou Q.M., Jia R., Yang W.G. (2019). Effects of ultra-high pressure on the biochemical properties and secondary structure of myofibrillar protein from oratosquilla oratoria muscle. J. Food Process Eng..

[44] Li Z.Y., Wang J.Y., Zheng B.D., Guo Z.B. (2019). Effects of high pressure processing on gelation properties and molecular forces of myosin containing deacetylated konjac glucomannan. Food Chem..

[45] Wang M.Y., Chen X., Zou Y.F., Chen H.Q., Xue S.W., Qian C., Wang P., Xu X.L., Zhou G.H. (2017). High-pressure processing-induced conformational changes during heating affect water holding capacity of myosin gel. Int. J. Food Sci. Technol..

[46] Li Y., Kang Z., Sukmanov V., Ma H. (2021). Effects of soy protein isolate on gel properties and water holding capacity of low-salt pork myofibrillar protein under high pressure processing. Meat Sci..

[47] Lv M.C., Tan B.B., Yang R., Xu A.Q., Zhang J.J., Xu D.L., Yang W.G. (2020). Effects of high pressure on biochemical properties and structure of myofibrillar protein from *Tegillarca granosa*. Int. J. Food Sci. Technol..

[48] Cando D., Herranz B., Javier Borderias A., Moreno H.M. (2015). Effect of high pressure on reduced sodium chloride surimi gels. Food Hydrocoll..

[49] Mwindaace N.S., Lord R.C., Chen M.C., Tadahisa T., Issei H., Hiroatsu M., Takehiko M. (1975). Interpretation of the doublet at 850 and 830 cm^−1^ in the raman spectra of tyrosyl residues in proteins and certain model compounds. Biochemistry.

[50] Li X.Y., Mao L., He X.Y., Ma P.H., Gao Y.X., Yuan F. (2018). Characterization of β-lactoglobulin gels induced by high pressure processing. Innov. Food Sci. Emerg. Technol..

[51] Zhang Z., Yang Y., Tang X., Chen Y., You Y. (2015). Chemical forces and water holding capacity study of heat-induced myofibrillar protein gel as affected by high pressure. Food Chem..

[52] He L., Gao Y., Han L., Yu Q., Zang R. (2021). Enhanced gelling performance of oxhide gelatin prepared from cowhide scrap by high pressure-assisted extraction. J. Food Sci..

[53] Kuan Y.H., Nafchi A.M., Huda N., Ariffin F., Karim A.A. (2016). Comparison of physicochemical and functional properties of duck feet and bovine gelatins. J. Sci. Food Agric..

[54] Xu M.Q., Wei L.X., Xiao Y.C., Bi H.T., Yang H.X., Du Y.Z. (2017). Physicochemical and functional properties of gelatin extracted from yak skin. Int. J. Biol. Macromol..

[55] Ellman G.L. (1959). Tissue sulfhydryl groups. Arch. Biochem. Biophys..

